# Supplementary material for: MetaRibo-Seq measures translation in microbiomes
Source: Nat Commun. 2020 Jun 29;11:3268. doi: 10.1038/s41467-020-17081-z (PMC7324362; doi:10.1038/s41467-020-17081-z)
Supplement: Supplementary file 10 — Supplementary Data 7 [file 41467_2020_17081_MOESM10_ESM.zip › File2/Confidence_High_Taxonomy/360180_out.krona.html]

Javascript must be enabled to view this page.

members
magnitude
magnitudeUnassigned
count
unassigned
taxon
rank

360180\_out

6

superkingdom
2
6

6
1239
phylum

class
186801
6

order
186802
6

1
541000
family

genus
216851
1

species

SRS143780\_contig\_number\_10771
1971605
1

5
31979
family

genus
1485
5

species
411486
5

SRS017821\_contig\_number\_7125SRS019068\_contig\_number\_111148SRS024435\_contig\_number\_contig-100\_1040.248931SRS063985\_contig\_number\_31201SRS098827\_contig\_number\_12923
